# A Phosphorus-Containing Polyamide Oligomer as a Synergist for Flame-Retardant PA66/GF Composites: Concurrent Improvements in Flame Retardancy and Electrical Safety

**DOI:** 10.3390/polym18212706

**Published:** 2026-11-01

**Authors:** Xing Zhang, Lirong Xie, Zhongliang Zhang, Guanghao Guo, Zhichao Zhen, Xuran Liu, Dan Huang

**Affiliations:** 1 College of Materials Engineering, North China Institute of Aerospace Engineering, Langfang 065000, China; xingzhang202506@163.com (X.Z.); xlr159159@163.com (L.X.); m13964452183@163.com (Z.Z.); huaqiangfan508@gmail.com (G.G.); 2 National Engineering Research Center of Engineering and Ecological Plastics, Technical Institute of Physics and Chemistry, Chinese Academy of Sciences, Beijing 100190, China; zhenzc@mail.ipc.ac.cn

**Keywords:** PA66/GF composites, halogen-free flame retardancy, phosphorus-containing polyamide oligomer, flame-retardant synergist, electrical safety

## Abstract

As electronics become smaller and more power-dense, glass-fiber-reinforced polyamide 66 (PA66/GF) must combine halogen-free flame retardancy with electrical safety, including a glow-wire ignition temperature (GWIT) ≥ 775 °C and a comparative tracking index (CTI) ≥ 600 V. Conventional low-molecular-weight flame retardants often require loadings above 15 parts by mass, impairing mechanical and electrical properties. Here, a phosphorus-containing polyamide oligomer (DOPO–MA–oligomerPA6) was synthesized via a DOPO–MAH intermediate from 9,10-dihydro-9-oxa-10-phosphaphenanthrene-10-oxide (DOPO) and maleic anhydride (MAH), followed by polymerization with ε-caprolactam, then melt-blended with aluminum diethylphosphinate (ADP) and melamine polyphosphate (MPP) in PA66/GF. Its oligo-PA6 backbone provides good compatibility with PA66, while its low molecular weight (Mn = 10,984 g mol^−1^; PDI = 2.569) favors dispersion. The phosphorus groups enhance the efficiency of the ADP/MPP system. With 4 parts oligomer and a total flame-retardant loading of 14 parts by mass per 100 parts PA66/GF, the composite achieved UL-94 V-0 rating, a limiting oxygen index (LOI) of 36.1%, a GWIT of 800 °C, and a CTI of 600 V. Its heat deflection temperature, tensile strength, notched impact strength, and flexural modulus were 178.8 °C, 95.0 MPa, 8.7 kJ m^−2^, and 8984 MPa, respectively. Compared with the conventional 20-part system, total additive loading decreased by 30%, with simultaneous improvements in flame retardancy, electrical safety, and mechanical properties. This offers a scalable route to high-performance PA66/GF composites.

## 1. Introduction

Polyamide 66 (PA66) is widely used in aerospace, automotive, electrical and electronic, and medical applications because of its mechanical strength, heat resistance, and fatigue resistance [1,2,3]. Glass fibers (GF) are commonly incorporated to improve load-bearing capacity and dimensional stability [3]. However, GF can also promote flame spread through the “wicking effect”: molten polymer is transported along the fibers, supplying fuel to the flame. The fiber–matrix interface and the limited contribution of GF to char formation further complicate the design of flame-retardant PA66/GF composites [4,5,6,7,8]. An effective formulation must therefore improve fire performance without unduly compromising interfacial integrity and mechanical properties.

The ongoing miniaturization of electrical equipment and the increase in its power density impose additional requirements on insulating components. In addition to a UL-94 V-0 rating, applications may require a GWIT of at least 775 °C and a CTI of at least 600 V [9,10,11]. These parameters reflect different aspects of material performance. Glow-wire ignition is influenced by thermal decomposition, char formation, and the release of combustible volatiles, whereas tracking resistance may be affected by surface chemistry, conductive residues, and additive migration [12,13,14]. Consequently, a higher LOI or an improved UL-94 rating does not necessarily translate into improved electrical safety. High additive loadings may also create agglomerates and interfacial defects that adversely affect electrical and mechanical properties [11,15,16,17].

Halogen-free flame-retardant systems based on phosphorus, nitrogen, silicon, and inorganic hydroxides have attracted considerable attention for PA66/GF [18]. ADP combined with MPP is a representative commercial system. Its flame-retardant action can involve gas-phase inhibition and the formation of a protective condensed-phase residue [19,20,21]. Nevertheless, high additive loadings are often required, and poor compatibility or additives can affect processability, mechanical properties, and electrical safety. Improving the efficiency of ADP/MPP at lower loadings is therefore of practical interest [22,23].

Synergists can enhance the effectiveness of a principal flame retardant by influencing decomposition and char formation at relatively low loadings [24]. The reactive P–H bond of 9,10-dihydro-9-oxa-10-phosphaphenanthrene-10-oxide (DOPO) makes it a useful building block for phosphorus-containing flame retardants. DOPO derivatives have been investigated in epoxy resins, polyesters, and polyamides [25,26,27,28,29]. For example, a DOPO-functionalized maleic anhydride copolymer has been reported to improve fiber–matrix interfacial adhesion in glass-fiber-reinforced polyamide [4]. However, compatibility, thermal processing, and electrical safety must also be considered when selecting a synergist for PA66/GF [30,31].

An oligomeric carrier may improve additive dispersion and interfacial interactions because of its structural affinity with a polyamide matrix [32,33]. In a related approach, low-molecular-weight nylon was used to coat melamine cyanurate (MCA), reducing agglomeration in PA66 and improving flow and mechanical performance at the same MCA loading [34]. This observation suggests that polyamide oligomers could provide a useful scaffold for phosphorus-containing synergists. Helmy et al. [35] synthesized a phosphorus- and nitrogen-containing macromolecular flame retardant (PN) and prepared PA6 composites containing 1–10 wt% PN. Their results showed that incorporating 1 wt% and 10 wt% PN enabled the materials to achieve UL-94 V-2 and V-0 ratings, respectively. Glow-wire testing further demonstrated that the phosphorus-containing macromolecular flame retardant influenced the ignition behavior of PA6. In addition, Fu et al. [16] employed a phosphorus–silicon synergistic strategy to develop flame-retardant reinforced PA66 composites with improved electrical safety. With the optimal formulation, the composites not only achieved a UL-94 V-0 rating but also exhibited a 25 °C increase in the glow-wire ignition temperature (GWIT) and a 225 V increase in the comparative tracking index (CTI) compared with conventional phosphorus–nitrogen flame-retardant systems, highlighting the potential of synergistic strategies to improve GWIT and CTI simultaneously. Regarding the underlying mechanisms, Ye et al. [36] investigated glass-fiber-reinforced PA6 containing organophosphine flame retardants. They reported that glass-fiber melting at the high temperatures generated by electrical arcing and the subsequent progressive formation of conductive pathways along the tracking region constituted one of the key mechanisms responsible for failure in CTI testing. From an industrial application perspective, Liu et al. [37] achieved a CTI of 600 V and a GWIT of 725 °C in a halogen-free flame-retardant PA66/GF system, providing a useful benchmark for the present study. Although phosphorus-containing oligomers have previously been investigated for flame retardancy in polyamides, most studies have focused on unreinforced PA6, employed phosphorus-containing components as the primary flame retardants, or primarily evaluated UL-94 ratings and limiting oxygen index (LOI) values, with relatively few studies simultaneously examining GWIT and CTI. Systematic research is still lacking on the design of phosphorus-containing oligomers as synergists for glass-fiber-reinforced PA66 composites that, at low total additive loadings, simultaneously achieve a UL-94 V-0 rating, a GWIT of at least 775 °C, and a CTI of at least 600 V.

In summary, synergistic strategies that provide good additive-matrix compatibility, require low additive loadings, and simultaneously enhance flame retardancy and electrical safety in PA66/GF systems are still lacking. To address this gap, a novel phosphorus-containing polyamide oligomer, DOPO–MA–oligomerPA6, was designed and synthesized via a one-pot process involving the addition reaction of DOPO with maleic anhydride (MAH), followed by ring-opening polymerization with ε-caprolactam (ε-CPL). The resulting oligomer was subsequently combined with PA66, chopped glass fibers, and ADP/MPP through melt blending to prepare flame-retardant PA66/GF composites. The oligomeric polyamide segments of the synergist are expected to improve its compatibility with the PA66 matrix. With only 4 parts by mass of the synergist and a total flame-retardant additive loading of 14 parts by mass per 100 parts PA66/GF, the composite achieved a UL-94 V-0 rating, a GWIT of 800 °C, and a CTI of 600 V, demonstrating favorable flame retardancy and electrical safety. Furthermore, the effects of the synergist on the mechanical properties, thermal properties, flame retardancy, and electrical safety of the composites were systematically evaluated. This study provides experimental evidence for the development of flame-retardant PA66/GF composites with low additive loadings and balanced overall performance, while offering a potential pathway toward industrial applications.

## 2. Materials and Methods

### 2.1. Materials

DOPO (analytical grade, 99.7%) and ε-caprolactam (industrial grade) were purchased from Shanghai Aladdin Biochemical Technology Co., Ltd. (Shanghai, China). MAH (analytical grade, 99.5%) was obtained from Sinopharm Chemical Reagent Co., Ltd. (Shanghai, China). Deionized water was prepared in the laboratory. PA66 resin (EPR27) was supplied by Pingdingshan Shenma Engineering Plastics Co., Ltd. (Pingdingshan, China). Aluminum diethylphosphinate (ADP; Exolit^®^ OP series) was obtained from Clariant (Muttenz, Switzerland); melamine polyphosphate (MPP) was supplied by Sichuan Jingshida Technology Co., Ltd. (Meishan, China); and chopped glass fibers (GF; T435N) were supplied by Taishan Fiberglass Inc. (Tai’an, China).

### 2.2. Synthesis of DOPO–MA–oligomerPA6

**Figure 1:**
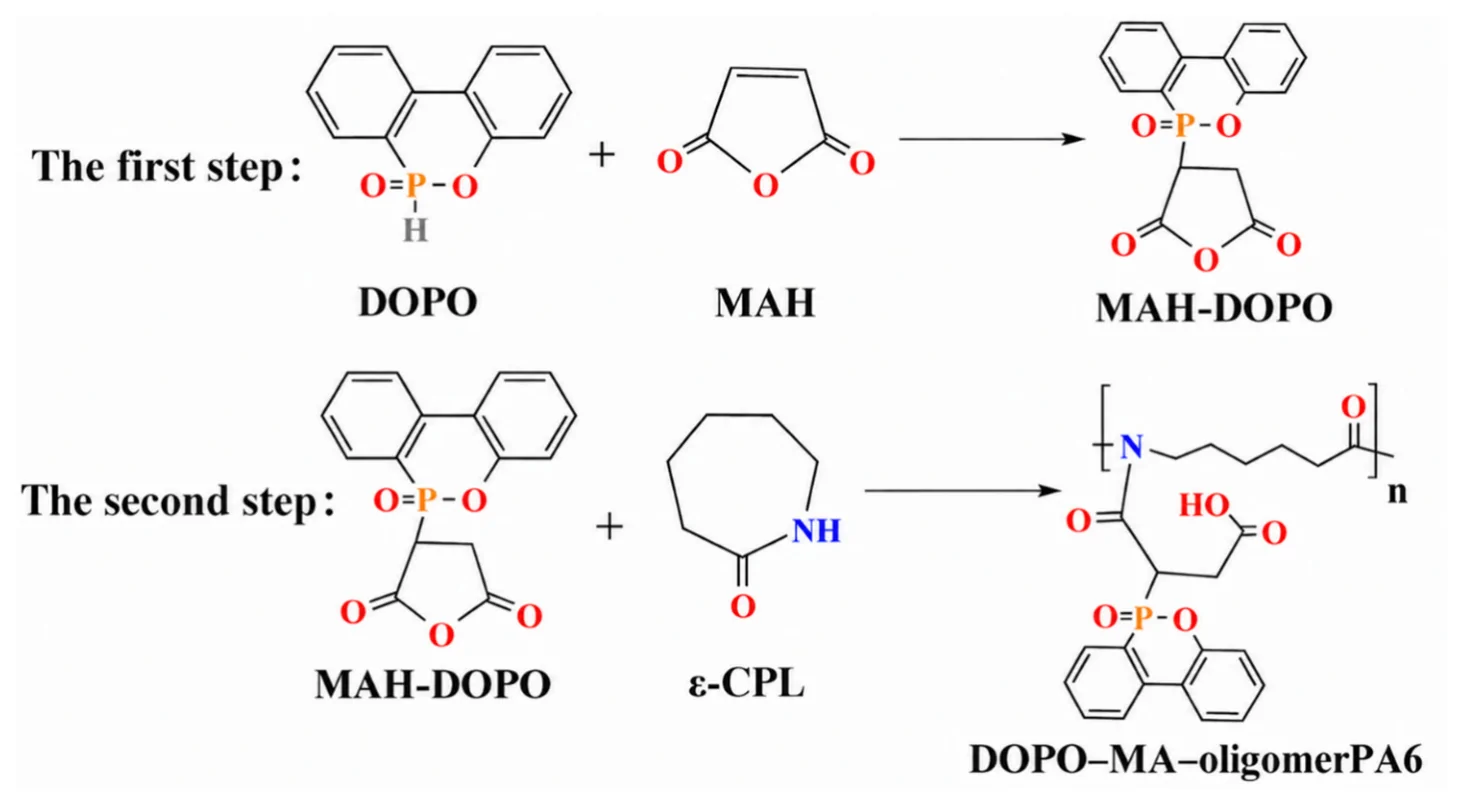
Synthetic route to DOPO–MA–oligomerPA6.

DOPO–MA–oligomerPA6 was synthesized via a one-pot method. First, the reactive P–H bond of DOPO underwent a nucleophilic addition reaction with the C=C double bond of maleic anhydride (MAH), yielding the DOPO–MAH intermediate. Subsequently, the intermediate reacted with ε-caprolactam (ε-CPL) through ring-opening polymerization, ultimately producing the target product, DOPO–MA–oligomerPA6. First, DOPO (1 mol) and MAH (1 mol) were placed in a dry 500 mL reaction vessel under nitrogen. The mixture was heated to 120 °C (10 °C/h) and stirred as a solvent-free melt for 8 h. Next, ε-CPL (1 mol) was added, and stirring and nitrogen protection were resumed. The temperature was raised to 220 °C at 10 °C h^−1^. At 220 °C, the nitrogen supply was stopped, and a mild vacuum(2 kPa) was applied for 1 h. The temperature was then increased to 250 °C (10 °C/h), and the reaction continued under high vacuum (<100 Pa) for 8 h. After the reaction was completed and the mixture was cooled, residual ε-caprolactam and other water-extractable components were removed by extraction with boiling water. The product was then collected by vacuum filtration and dried under vacuum at 50 °C to constant weight, yielding DOPO–MA–oligomerPA6. The product was not subjected to recrystallization. The synthesis yield was 50.8%, and the number-average degree of polymerization (X_n_) was 25.70 [38,39,40,41]. The synthetic route is illustrated in Figure 1.

### 2.3. Preparation of Flame-Retardant PA66/GF Composites

**Figure 2:**
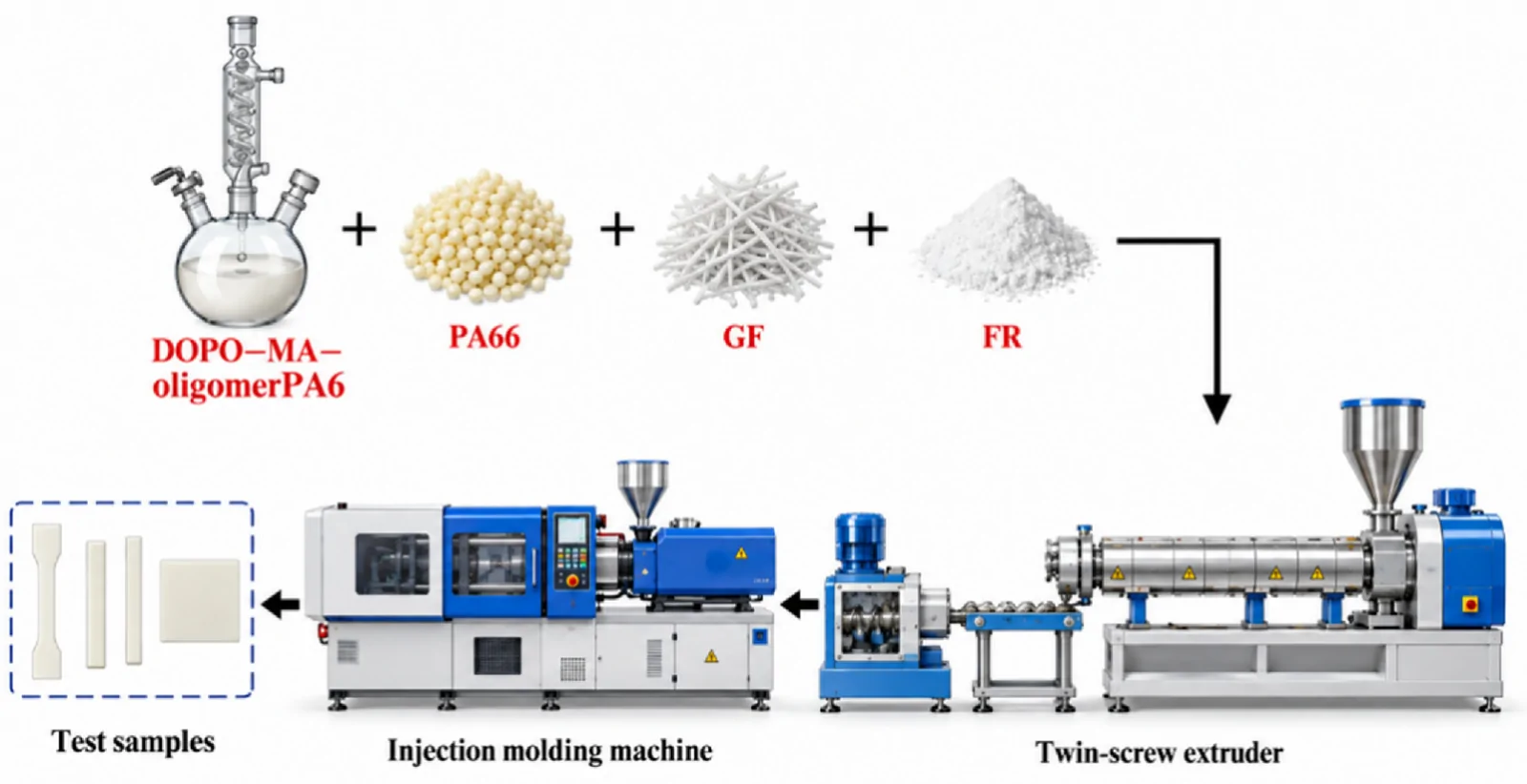
Schematic illustration of the preparation of flame-retardant PA66/GF composites.

PA66, GF, ADP/MPP, and DOPO–MA–oligomerPA6 were vacuum-dried at 80 °C for 12 h, mixed in the proportions listed in Table 1, and pelletized using a twin-screw extruder (LSF-26, Labtech Engineering Co., Ltd., Samut Prakan, Thailand). The screw and feed speeds were 170 and 25 rpm, respectively. The eight-barrel temperatures were 250, 255, 260, 260, 260, 255, 250, and 245 °C. Standard test specimens were injection molded (SSF320-G, Ningbo Shuang Sheng Plastic Machinery Co., Ltd., Ningbo, China) at barrel temperatures of 280, 255, and 250 °C, a mold temperature of 90 °C, and an injection pressure of 80 MPa. For sheets, the pellets were vacuum-dried at 80 °C for 24 h, preheated at 270 °C for 10 min in a hot press (LP20-B, Labtech Engineering Co., Ltd., Samut Prakan, Thailand), pressed at 3 MPa for 10 min, cold-pressed for 6 min, and cooled. The preparation process is shown in Figure 2.

### 2.4. Characterization Methods

Structural characterization: ^1^H NMR spectra were recorded using a Bruker AVANCE III HD 300 MHz (Bruker BioSpin GmbH, Rheinstetten, Germany) spectrometer with deuterated trifluoroacetic acid as the solvent and tetramethyl silane as the chemical-shift reference. FT-IR spectra were acquired on an INVENIO S spectrometer over (Bruker Optik GmbH, Ettlingen, Germany) 4000–400 cm^−1^ at a resolution of 4 cm^−1^. Molecular weights were determined by gel permeation chromatography (GPC; Waters e2695, Waters Corporation, Milford, MA, USA) using HFIP containing 0.1% sodium trifluoroacetate as the mobile phase and PMMA standards for calibration.

Mechanical properties were measured using a WDW-10 universal testing machine (Shanghai Songdun Instrument Manufacturing Co., Ltd., Shanghai, China). Tensile and flexural properties were determined in accordance with GB/T 1040.2-2022 [42] and GB/T 9341-2008 [43], respectively. Charpy impact properties were determined using an XJJD-5 electronic Charpy impact testing machine (Chengde Zongchi Testing Instrument Co., Ltd., Chengde, China) in accordance with GB/T 1043.1-2008 [44]. For each test group, five specimens were tested. After excluding the maximum and minimum values, the arithmetic mean of the results obtained from the remaining three specimens was reported as the final result, and the error bars represented the standard deviation (SD). Morphology was examined by field-emission scanning electron microscopy (FESEM; SU8020, Hitachi, High-Tech Corporation, Tokyo, Japan) at an accelerating voltage of 5 kV and a working distance of approximately 8 mm. Samples were mounted with conductive adhesive and sputter-coated with gold for 60 s using a JFC-1600 coater (JEOL Ltd., Akishima, Tokyo, Japan).

Thermal performance characterization testing: thermal deformation temperature measurement (HDT; VICAT voltage 220 V, Cangzhou Xinxing Testing Instrument Co., Ltd., Cangzhou, China): test according to GB/T1634.2-2019 [45] standard, spline size (length × width × thickness): 80 mm × 10 mm × 4 mm (test pressure is 1.82 MPa). For each test group, five specimens were tested. After excluding the maximum and minimum values, the arithmetic mean of the results obtained from the remaining three specimens was reported as the final result, and the error bars represented the standard deviation (SD); thermogravimetric analysis test (TGA, Hitachi STA200, Hitachi High-Tech Science Corporation, Tokyo, Japan): test according to the standard of GB/T33047.1-2016 [46], under the protection of N2, heating rate (10 °C/min), temperature range (20~800 °C); differential scanning calorimetry test (DSC, Discovery DSC 2500, TA Instruments, New Castle, DE, USA): according to GB/T19466.3-2004 [47] standard test, under the protection of N2, heating/cooling rate (10 °C/min), temperature range (20~300 °C).

Flame-Retardancy Testing: The UL-94 vertical burning test (TESTech Instrument (Suzhou) Technologies Co., Ltd., Suzhou, China) was conducted in accordance with the GB/T 2408-2021 [48] standard using specimens with dimensions of 130 mm × 13 mm × 1.6 mm (length × width × thickness). A flammability rating was assigned only when all five replicate specimens met the corresponding classification criteria. Cone calorimetry (VOUCH6810, Suzhou Yangyi Vouch Testing Technology Co., Ltd., Suzhou, China) was performed according to ISO 5660-1:2015 [49] on 100 × 100 × 3 mm^3^ specimens at a heat flux of 35 kW m^−2^. The limiting oxygen index (LOI, TESTech Instrument (Suzhou) Technologies Co., Ltd., Suzhou, China) was measured according to GB/T 2406.2-2009 [50] using 150 × 6 × 3 mm^3^ specimens; the arithmetic mean of five specimens was reported. The LOI was calculated from the oxygen [*V*(O_2_)] and nitrogen [*V*(N_2_)] flow rates as follows:

(1) $$\mathrm{LOI}=\frac{V(O_{2})}{V\left(O_{2}\right)+V(N_{2})}\times 100$$

Electrical Safety Testing: The glow-wire ignition temperature (GWIT) test (K-R5169, Suzhou Kaiteer Instrument & Equipment Co., Ltd., Suzhou, China) was conducted in accordance with the GB/T 5169.13-2024 [51] standard using specimens with dimensions of 60 mm × 60 mm × 1.5 mm (length × width × thickness). The GWIT was determined by adding 25 °C to the highest test temperature at which all three specimens of the specified thickness met the prescribed test criteria, or by adding 30 °C for the specified higher-temperature range. The comparative tracking index (CTI) test (K-R4207, Suzhou Kaiteer Instrument & Equipment Co., Ltd., Suzhou, China) was conducted in accordance with the GB/T 4207-2022 [52] standard using specimens with dimensions of 60 mm × 60 mm × 3 mm (length × width × thickness). Each specimen was subjected to 50 drops of the test solution per test. The CTI was determined as the highest test voltage at which all five replicate specimens met the prescribed test criteria.

## 3. Results and Discussion

### 3.1. Structure of DOPO–MA–oligomerPA6

**Figure 3:**
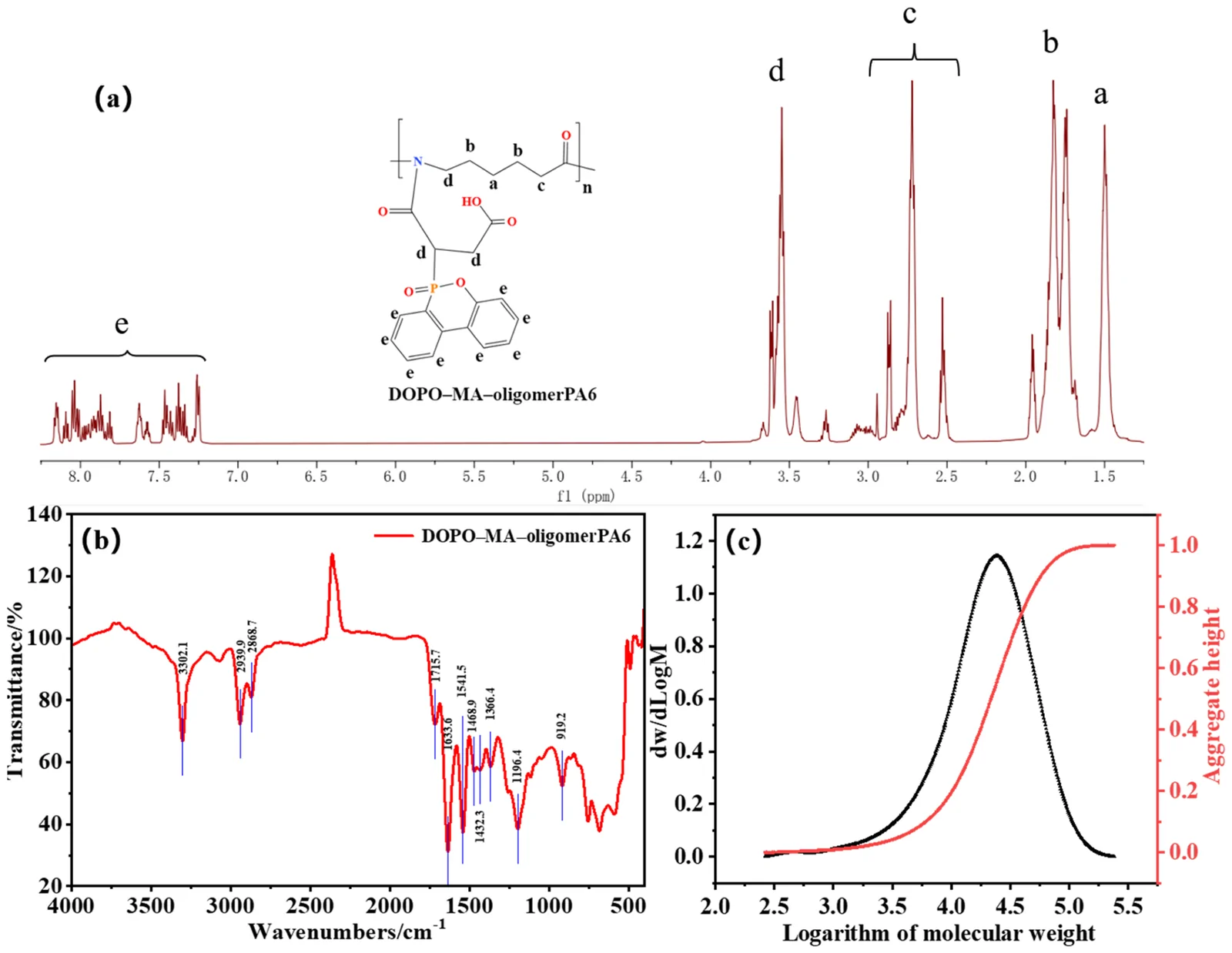
(**a**) Proton nuclear magnetic resonance (^1^H NMR) spectrum, (**b**) Fourier-transform infrared (FT-IR) spectrum, and (**c**) gel permeation chromatography (GPC) molecular-weight distribution of DOPO–MA–oligomerPA6.

As illustrated in Figure 1, DOPO was reacted with the C=C bond of MAH through its reactive P–H group, and the resulting intermediate was subsequently reacted with ε-CPL to obtain DOPO–MA–oligomerPA6. In the ^1^H NMR spectrum (Figure 3a), the multiplet at δ 7.25–8.25 ppm (signal e) is assigned to the aromatic protons of DOPO [4,38,41,53,54,55]. The series of signals in the range of δ 1.2–3.7 ppm (signals a–d) are attributed to the methylene and methine protons of the PA6 segments and MAH units [4,38,41,53,54,55]. These proton signals can be further assigned as follows: signal a corresponds to the aliphatic methylene groups (–CH_2_–) in the middle of the PA6 segments; signal b is attributed to the methylene groups (–CH_2_–) adjacent to those corresponding to signal a; signal c is assigned to the methylene groups adjacent to carbonyl groups (–CH_2_–C(=O)–); and signal d is associated with the methylene groups adjacent to amide nitrogen atoms (–CH_2_–N–), the methylene groups adjacent to carboxyl groups (–CH_2_–COOH) in the MAH units, and the methine groups bonded to phosphorus atoms and adjacent to carboxyl groups (–CH(P)–) in the side chains. Comparison of these signals reveals that signal d is shifted significantly downfield (δd > δc > δb), primarily due to differences in the electronic effects of neighboring groups. Signal d is influenced by the relatively electronegative amid nitrogen atoms and the strong electron-withdrawing inductive effect of the phosphorus-containing groups (P=O). These effects lead to pronounced deshielding, resulting in a substantially higher chemical shift than those of signals c and b, which are mainly influenced by the carbonyl groups along the polymer backbone. Furthermore, although some methylene signals are theoretically expected to exhibit triplet splitting, the actual spectrum shows relatively complex peak shapes in this region. For signal c (–CH_2_–C(=O)–), a triplet is theoretically expected due to coupling with two protons on the adjacent carbon atom. However, the signal may be broadened by variations in the local chemical environments of the oligomer and overlapping resonances. The complexity of the signal d region arises from the superposition of multiple signals. On the one hand, the quadrupolar relaxation effect of amide nitrogen (^14^N) contributes to the broadening of the adjacent –CH_2_– signals, thereby obscuring their fine splitting patterns. On the other hand, the –CH(P)– proton undergoes spin–spin coupling with phosphorus (^31^P), resulting in multiplet splitting. The combined effects give rise to broad and complex multiplet patterns in this region. These results further confirm the ring-opening polymerization of ε-caprolactam and the formation of the target DOPO–MA–oligomerPA6 structure [4,38,41,53,54,55].

The FT-IR results (Figure 3b and Table 2) further support the proposed structure of the product. Since amide groups are key structural features of the PA6 segments in the target product, the analysis focuses primarily on the characteristic absorption bands associated with amide groups. The absorption peak near 3302 cm^−1^ is assigned to N–H stretching vibrations, while the peak at 1633 cm^−1^ is mainly attributed to the amide I band (C=O stretching vibrations). Notably, the absorption peak at 1541 cm^−1^ not only includes the amide II band (coupled N–H in-plane bending and C–N stretching vibrations) but also overlaps with the aromatic C=C stretching vibrations due to the incorporation of the DOPO structure. Together with the methylene C–H stretching vibrations at 2939 and 2868 cm^−1^ and the CH_2_ bending vibration at 1468 cm^−1^, these characteristic absorption peaks support the presence of amide structures and aliphatic chain segments in the product. In addition, the broad absorption band in the range of 1196 cm^−1^ is mainly attributed to P=O, P–O–C, and aromatic C–O stretching vibrations, which may also overlap with some amide III band vibrations. The absorption near 1432 cm^−1^ may be associated with vibrations of the aromatic ring framework and P–aryl groups in DOPO, while the peak at 919 cm^−1^ is assigned to P–O–Ar-related vibrations in the DOPO structure. The band at 1715.7 cm^−1^ is mainly attributed to the C=O stretching vibration of carboxyl groups, consistent with the –COOH group shown in the proposed final structure. The band at 1366.4 cm^−1^ is assigned to CH_2_ deformation and coupled chain-segment vibrations. These characteristic absorption bands collectively support the retention of the DOPO-related phosphorus-containing structure. Due to the complexity of the system, some absorption bands exhibit significant overlap of multiple vibrational signals; nevertheless, the overall spectral features remain consistent with the expected structure. Furthermore, no obvious characteristic P–H absorption is observed near 2400 cm^−1^, and no distinct anhydride C=O doublet is detected near 1850 and 1775 cm^−1^, which is consistent with the expected P–H addition and anhydride ring-opening reactions. Overall, the FT-IR absorption characteristics of the product are generally consistent with the expected functional group composition of DOPO–MA–oligomerPA6 [4,38,41,53,54,55].

The GPC results (Figure 3c and Table 3) show that the product has a number-average molecular weight (M_n_) of 10,984 g/mol, a weight-average molecular weight (M_w_) of 28,223 g/mol, and a polydispersity index (PDI) of 2.569, indicating that the product is a low-molecular-weight oligomer with a narrow molecular weight distribution. Collectively, these results confirm the successful synthesis of the target DOPO–MA–oligomerPA6. Its moderate oligomer chain length and narrow molecular weight distribution are favorable for achieving uniform dispersion in the PA66 matrix.

### 3.2. Properties of the Flame-Retardant PA66/GF Composites

#### 3.2.1. Mechanical Properties

Table 4 summarizes the mechanical properties. Incorporating GF increased the tensile strength from 77.1 to 120.6 MPa, the flexural modulus from 2500 to 8903 MPa, and the notched impact strength from 3.7 to 8.7 kJ m^−2^. Relative to P0, the tensile strengths of P1, P2, and P3 decreased from 120.6 MPa to 110.3, 95.1, and 90.2 MPa, corresponding to decreases of 8.5%, 21.1%, and 25.2%, respectively. Their notched impact strengths decreased from 8.7 to 8.4, 8.1, and 7.9 kJ m^−2^ (decreases of 3.4%, 6.9%, and 9.2%), while the unnotched impact strengths decreased from 52.2 to 38.6, 36.2, and 35.7 kJ m^−2^ (26.1%, 30.7%, and 31.6%). The flexural modulus initially increased to 9610 MPa for P1, then decreased to 9058 and 8597 MPa for P2 and P3. At a fixed ADP/MPP loading of 10 parts by mass, adding the oligomer yielded a tensile strength of 99.8 MPa, a flexural modulus of 9321 MPa, an elongation at break of 5.3%, and notched and unnotched impact strengths of 8.5 and 38.5 kJ m^−2^ for P4. At the highest oligomer loading (P8), tensile strength and flexural modulus were 93.5 and 8800 MPa, whereas elongation at break increased to 6.0%; notched and unnotched impact strengths were 8.8 and 38.8 kJ m^−2^. Thus, relative to P2 and P3, P8 showed similar tensile strength but greater elongation and notched impact strength. The oligomer-containing formulations provide a favorable balance of stiffness and toughness compared with simply increasing ADP/MPP loading.

**Figure 4:**
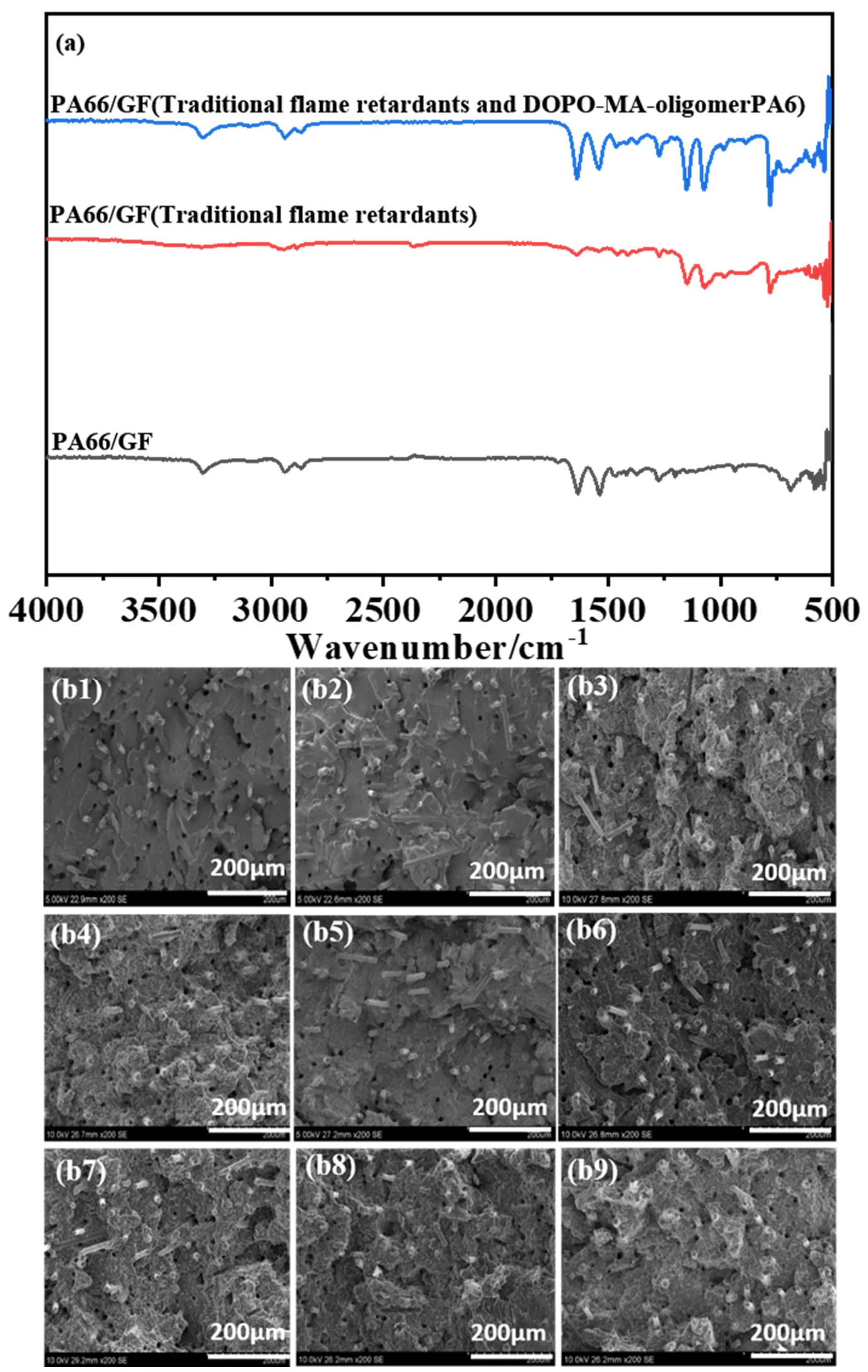
(**a**) Fourier-transform infrared (FT-IR) spectra of PA66, PA66/GF, and the flame-retardant composites; (**b1**–**b9**) scanning electron microscopy (FESEM) images of the fracture surfaces of P0, P1, P2, P3, P4, P5, P6, P7, and P8, respectively.

Figure 4a and Table 5 summarize the FT-IR characteristics of different PA66/GF composites. All samples exhibit a characteristic absorption band corresponding to amide N–H stretching vibrations at approximately 3300 cm^−1^. The absorption bands at approximately 1633 and 1541 cm^−1^ are assigned to the amide I and amide II bands, respectively, which are mainly associated with C=O stretching vibrations and coupled N–H in-plane bending and C–N stretching vibrations. The absorption bands at approximately 2940 and 2870 cm^−1^ are attributed to aliphatic CH_2_ stretching vibrations. The positions of these characteristic absorption bands remain generally consistent across the samples, indicating that the main amide structural features of PA66 are retained after the incorporation of conventional flame retardants and DOPO–MA–oligomerPA6. Compared with PA66/GF, the flame-retardant composites exhibit noticeable changes in the shapes of the absorption bands within the 1000–1300 cm^−1^ region, which may be associated with P–O and P=O vibrations of the phosphorus-containing components. However, the overlapping contributions from Si–O vibrations of GF and absorption bands of the PA66 matrix should also be considered. Overall, the FT-IR results indicate that the composites retain the main structural characteristics of PA66, suggesting that the chemical structure of the PA66 backbone remains largely unchanged [5,6].

The FESEM fracture-surface morphologies (Figure 4(b1–b9)) further support the above interpretation. When 10 parts of the conventional flame retardants were added (Figure 4(b2)), the fracture surface was relatively uniform, with no obvious large agglomerates. However, when the loading of the conventional flame retardants was increased to 15 and 20 parts (Figure 4(b3,b4)), the fracture surfaces became noticeably rougher, accompanied by more protruding particles, agglomerates, and interfacial defects, indicating that high additive loadings adversely affect the dispersion of the flame retardants in PA66/GF and deteriorate the fiber–matrix interface. In contrast, after the incorporation of DOPO–MA–oligomerPA6 (Figure 4(b5–b9)), the composites retained a relatively uniform morphology similar to that of Figure 4(b2), with no obvious agglomeration or phase separation, suggesting that DOPO–MA–oligomerPA6 does not impair the compatibility of the system and may instead help maintain the uniform dispersion of the conventional flame retardants at lower loadings. Therefore, the synergistic system can achieve the required flame-retardant and electrical-safety performance without increasing the conventional flame-retardant loading to 15–20 parts, thereby avoiding the interfacial deterioration and mechanical-property losses associated with high additive loadings. Taken together, the FT-IR and FESEM results indicate that the PA66 backbone remains intact, favorable interactions exist between the synergist and the matrix, and the individual components are relatively uniformly dispersed; these combined effects enable the DOPO–MA–oligomerPA6-containing synergistic system to achieve a favorable balance between stiffness and toughness at a lower total additive loading, providing a feasible strategy for developing PA66/GF composites with high flame retardancy and enhanced electrical safety.

#### 3.2.2. Thermal Properties

**Figure 5:**
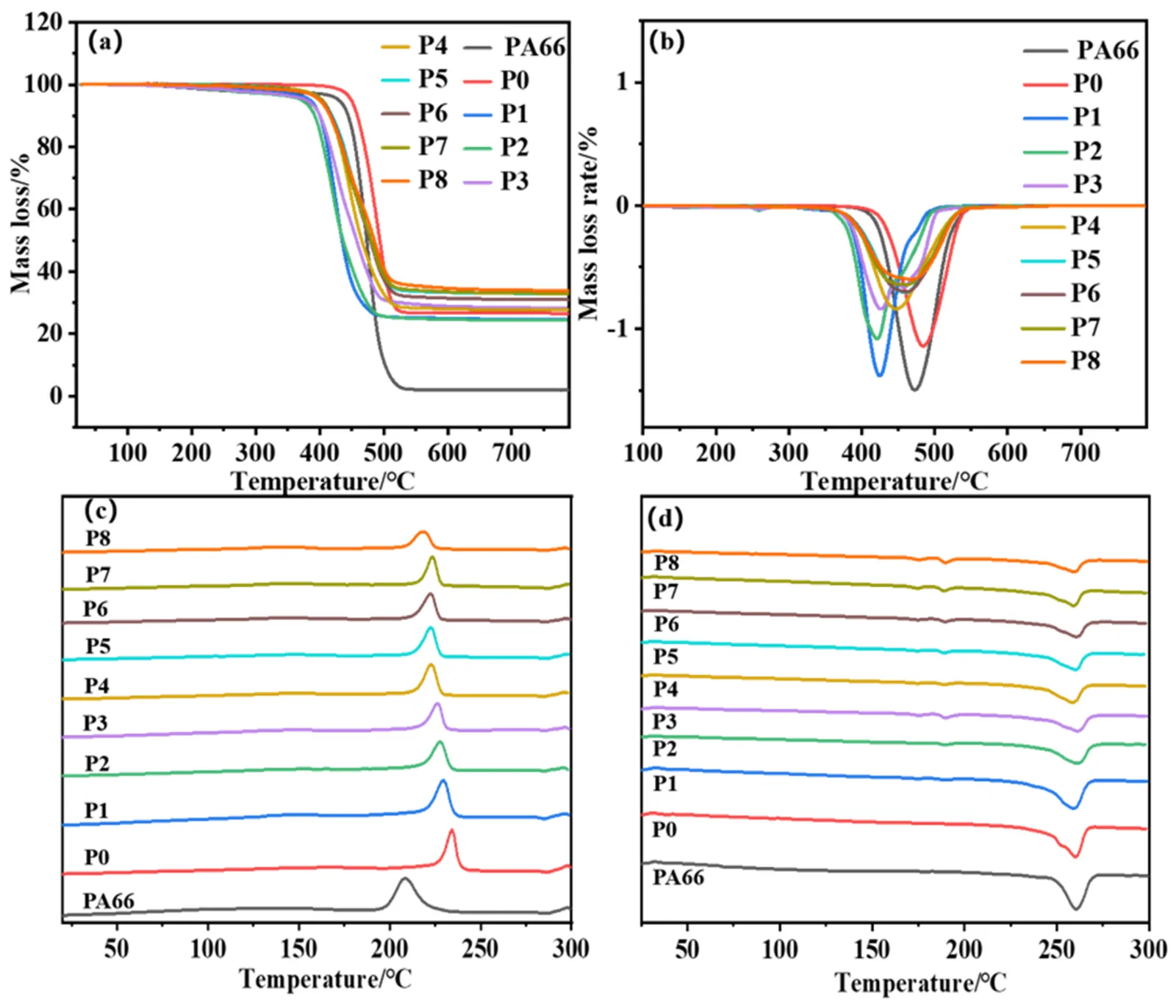
(**a**) Thermogravimetric analysis (TGA) and (**b**) derivative thermogravimetry (DTG) curves, (**c**) first-cooling crystallization curves, and (**d**) second-heating melting curves of PA66 and the PA66/GF composites.

To evaluate the effects of different components on the thermal properties of flame-retardant PA66/GF composites, DSC, TGA/DTG, and heat deflection temperature (HDT) measurements were performed on all samples (Figure 5 and Table 6). The results showed that GF, the ADP/MPP flame-retardant blend, and DOPO–MA–oligomerPA6 markedly affected the crystallization behavior, thermal decomposition behavior, and load-bearing performance of the composites at elevated temperatures.

Neat PA66 exhibited a crystallization peak temperature (*T_c_*) of 208.2 °C and a crystallinity of 23.3%. After the incorporation of 25 parts by mass of GF, the *T_c_* of P0 increased to 234.5 °C, and its crystallinity increased to 29.0%. Meanwhile, its HDT increased substantially from 70.3 to 215.7 °C. These results indicated that GF promoted PA66 crystallization by providing heterogeneous nucleation sites and substantially improved the dimensional stability of the material at elevated temperatures by enhancing its rigidity and restricting molecular chain mobility. The TGA/DTG results also showed that the temperature at 5% mass loss (*T*_5%_) and the temperature at the maximum mass loss rate (*T_max_*) increased from 430.2 and 472.6 °C for neat PA66 to 449.3 and 484.0 °C for P0, respectively. The residual mass at 700 °C increased from 2.0% for neat PA66 to 26.7% for P0, indicating that GF incorporation contributed to the overall thermal stability of the system.

The incorporation of the conventional ADP/MPP flame-retardant blend markedly affected the crystallization behavior and HDT of the composites. With the addition of 10 parts by mass of ADP/MPP, the crystallinity of P1 increased slightly to 30.8%, compared with 29.0% for P0, suggesting that a small amount of the flame-retardant blend might have promoted heterogeneous nucleation. As the ADP/MPP content increased to 15 and 20 parts by mass, the crystallinity decreased to 26.3% for P2 and 19.3% for P3, respectively, indicating that higher flame-retardant contents hindered the regular arrangement of PA66 molecular chains and crystal growth. Meanwhile, the HDT decreased from 215.7 °C for P0 to values ranging from 136.9 to 142.3 °C for P1–P3, indicating that the incorporation of the flame-retardant blend reduced the ability of the composites to retain their stiffness under load at elevated temperatures.

The TGA/DTG results showed that although the incorporation of ADP/MPP decreased *T*_5%_ and *T_max_*, increasing its content reduced the maximum mass loss rate and increased the residual mass at elevated temperatures. These results suggested that ADP/MPP did not simply improve the initial thermal stability but instead promoted condensed-phase char formation through earlier decomposition, thereby slowing the subsequent main decomposition process. With the ADP/MPP content fixed at 10 parts by mass, the further incorporation of DOPO–MA–oligomerPA6 generally decreased *T_c_* and crystallinity in P4–P8. In particular, the crystallinity decreased from 18.3% for P4 to 11.3% for P8, indicating that the synergist inhibited PA66 crystal growth to some extent. Nevertheless, the HDT increased markedly, reaching 165.1–178.8 °C for P4–P7, with P7 exhibiting the highest value of 178.8 °C. These results suggested that the improvement in load-bearing performance at elevated temperatures did not depend on increased crystallinity but was more likely associated with the effects of the polyamide oligomer segments on interfacial adhesion and molecular chain mobility.

DOPO–MA–oligomerPA6 also substantially improved the thermal decomposition behavior of the system at elevated temperatures. As its content increased, *T_max_* generally increased for P4–P8, the maximum mass loss rate progressively decreased, and the residual mass at 700 °C generally increased. In particular, P8 exhibited a *T_max_* of 467.8 °C, a maximum mass loss rate of 0.601, and a residual mass of 34.1% at 700 °C. These results indicated that the synergist effectively slowed rapid thermal degradation during the main decomposition stage and promoted char formation at elevated temperatures.

Notably, the HDT of P8 decreased to 167.5 °C, representing a reduction of 11.3 °C (approximately 6.3%) relative to P7. However, it remained 25.2 °C (approximately 17.7%) higher than that of P1 without the synergist (142.3 °C) and also exceeded those of P2 and P3. These results indicated that the synergist substantially improved the HDT over a relatively broad range of addition levels, while the decrease observed for P8 represented a limited decline beyond a suitable addition level. The crystallinity decreased from 13.6% for P7 to 11.3% for P8, and the flexural modulus decreased slightly from 8984 to 8800 MPa. These changes suggested that an excessive oligomer content might have affected crystal perfection and stiffness retention, thereby adversely affecting the HDT. Nevertheless, *T_max_* and the residual mass at 700 °C remained relatively high for P8, indicating that its thermal decomposition stability did not deteriorate correspondingly. Variations in HDT could also have been influenced by interfacial conditions, specimen thermal history, and measurement variability. Therefore, the decrease observed for P8 could not be attributed entirely to a single factor.

Overall, GF primarily enhanced the crystallization ability, thermal stability, and HDT of PA66. ADP/MPP promoted earlier decomposition and char formation, whereas higher addition levels inhibited crystallization and reduced the HDT. Although DOPO–MA–oligomerPA6 further decreased crystallinity, it improved thermal stability during the main decomposition stage, increased the char yield at elevated temperatures, and markedly increased the HDT at suitable addition levels. Among the formulations investigated, P7 exhibited a favorable balance between thermal stability and HDT, whereas P8 was more effective in enhancing thermal decomposition stability and promoting char formation at elevated temperatures.

#### 3.2.3. Flame Retardancy

Table 7 presents the results of the UL-94 vertical burning and LOI tests for PA66 and its composites. Neat PA66 exhibited an LOI of 24.1%, which decreased to 21.4% for P0 after the incorporation of GF. Both samples exhibited melt dripping that ignited the cotton indicator, and neither achieved a UL-94 rating. These results indicate that although GF can improve the mechanical properties of the composites, its incorporation adversely affects their self-extinguishing behavior during combustion, consistent with the “wicking effect” observed in glass-fiber-reinforced polyamide systems. After the incorporation of ADP/MPP, the LOI increased with increasing ADP/MPP loading. The LOI values of P1, P2, and P3 were 28.5%, 33.6%, and 37.2%, respectively. Among these samples, both P2 and P3 achieved a UL-94 V-0 rating without melt dripping that ignited the cotton indicator, demonstrating that increasing the ADP/MPP loading effectively improved the flame retardancy of the composites.

At a fixed total ADP/MPP loading of 10 phr, the incorporation of DOPO–MA–oligo-merPA6 further altered the combustion behavior of the composites. With the addition of 1 phr of the synergist, P4 exhibited an LOI of 28.1%, showing no improvement compared with P1. However, its afterflame time was shortened, and melt dripping was eliminated, indicating that the effect of the synergist at a low loading was primarily reflected in reduced afterflame duration and improved dripping behavior. As the synergist loading increased from 2 to 5 phr, the LOI values of P5–P8 progressively increased from 31.4% to 39.2%, while the corresponding total afterflame time (t_1_ + t_2_) decreased from 11 s to 1 s. Notably, P6 achieved an LOI of 33.8% with a total flame-retardant additive loading of only 13 phr, comparable to that of P2 containing 15 phr of ADP/MPP. P7 exhibited an LOI of 36.1%, approaching that of P3 containing 20 phr of ADP/MPP, while reducing the total additive loading by 30%. P8 and P2 had the same total additive loading of 15 phr, but the LOI of P8 was 5.6 percentage points higher than that of P2. These comparisons demonstrate that, compared with simply increasing the ADP/MPP loading, incorporating an appropriate amount of DOPO–MA–oligomerPA6 enables higher LOI values and shorter afterflame times at comparable or lower total additive loadings.

**Figure 6:**
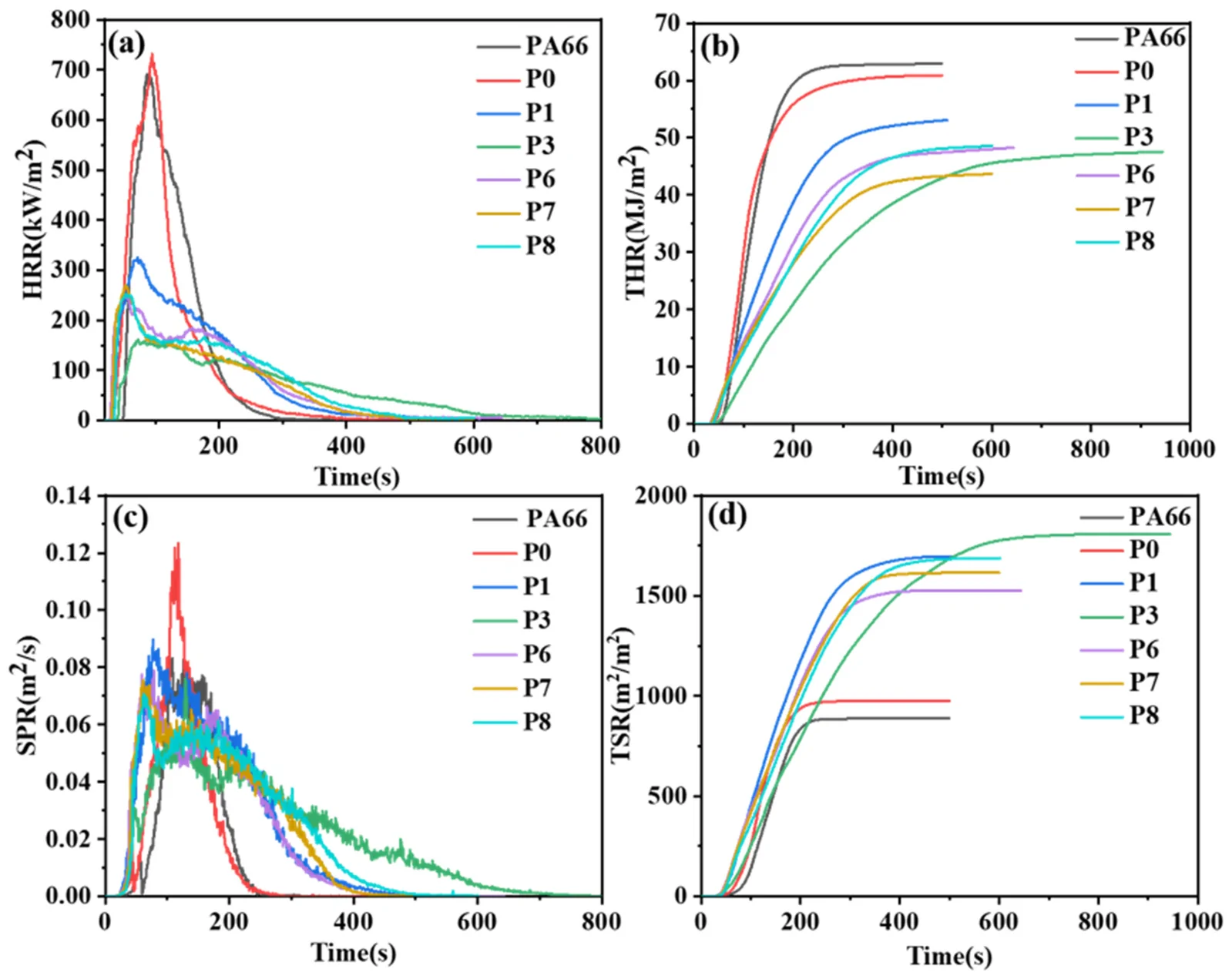
(**a**) Heat release rate (HRR), (**b**) total heat release (THR), (**c**) smoke production rate (SPR), and (**d**) total smoke release (TSR) of PA66 and the PA66/GF composites.

Cone calorimetry further revealed differences in the combustion behavior of the various formulations under sustained radiant heat exposure. As shown in Table 8 and Figure 6, P0 exhibited a peak heat release rate (pHRR) of 732 kW·m^−2^ and a total heat release (THR) of 61 MJ·m^−2^. After the addition of 10 phr of ADP/MPP, the pHRR of P1 decreased to 325 kW·m^−2^, representing a reduction of 55.6%, whereas its THR decreased by only 13.1%. These results indicate that, at this loading, ADP/MPP effectively suppressed the peak heat release but had a relatively limited effect on the cumulative heat release throughout the combustion process. When the ADP/MPP loading was increased to 20 phr, the pHRR of P3 further decreased to 165 kW·m^−2^, the time to peak heat release increased from 72 s for P1 to 121 s for P3, and the THR decreased to 48 MJ·m^−2^. These results suggest that increasing the loading of conventional flame retardants is beneficial for slowing heat release and reducing its peak intensity.

After the incorporation of the synergist, P6, P7, and P8 exhibited pHRR values of 252, 270, and 252 kW·m^−2^, respectively, representing reductions of 22.5%, 16.9%, and 22.5% compared with P1. Their corresponding THR values were 48, 44, and 49 MJ·m^−2^, respectively, all of which were lower than that of P1. Among these formulations, P7 exhibited the lowest THR, with reductions of 27.9% and 17.0% compared with P0 and P1, respectively, indicating its relatively strong ability to suppress cumulative heat release. However, the pHRR values of the synergistic formulations remained higher than that of P3, and their peak heat release occurred earlier. This indicates that the synergistic formulations were less effective than the high-loading ADP/MPP system in suppressing peak heat release and delaying its occurrence. Although P7 exhibited the lowest THR, its pHRR was slightly higher than those of P6 and P8. Therefore, the overall flame-retardant performance of different formulations cannot be evaluated based on a single parameter alone.

Increasing the synergist load did not lead to simultaneous improvements in all cone calorimetry parameters. When the synergist loading increased from 4 to 5 phr, P8 exhibited a further increase in LOI and a shorter afterflame time, whereas the THR increased from 44 MJ·m^−2^ for P7 to 49 MJ·m^−2^ for P8. These results indicate that increasing the synergist loading was more beneficial for improving the self-extinguishing behavior of the composites, as reflected by the LOI and vertical burning results, but did not further reduce the cumulative heat release under sustained radiant heat exposure. In addition, the time to ignition (TTI) values of P6–P8 ranged from 38 to 44 s, close to the 43 s recorded for P0, showing no consistent trend toward delayed ignition. Therefore, the main advantage of the synergist was reflected in the suppression of combustion after ignition rather than a continuous improvement in ignition resistance.

The residual mass after combustion provides additional information for understanding the changes in heat release. The residual mass fractions of P6–P8 were 35.54%, 39.06%, and 39.21%, respectively, all higher than that of P1 (31.23%). Notably, P7 achieved a residual mass fraction comparable to that of P3 with a total flame-retardant additive loading of 14 phr, while exhibiting a lower THR. This suggests that an appropriate synergist loading may favor the formation or retention of solid residues after combustion. However, because the residues also contained GF and inorganic components, the residual mass fraction cannot be directly equated with char yield, nor is it sufficient to determine the compactness of the char layer. Considering the phosphorus-containing structure and the thermogravimetric analysis results, the synergist may reduce heat release during combustion by modifying the thermal decomposition process, promoting condensed-phase residue formation, and acting in conjunction with ADP/MPP. Nevertheless, the specific reactive species in the gas phase and the structures formed in the condensed phase require further characterization.

The smoke release results also revealed differences among the evaluated parameters. As shown in Figure 6, the peak smoke production rate of P8 decreased to 0.0706 m^2^·s^−1^, approximately 42.8% lower than that of P0, indicating its ability to reduce the peak rate of smoke release during combustion. However, the flame-retarded samples generally exhibited higher total smoke production than P0, indicating that a reduction in the peak smoke production rate does not necessarily correspond to a simultaneous decrease in cumulative smoke production. Compared with P3, which contained a higher loading of conventional flame retardants, P6–P8 exhibited lower total smoke production. This suggests that the synergistic formulations offered a certain relative advantage in reducing cumulative smoke release while decreasing the total flame-retardant additive loading. Nevertheless, these results are insufficient to demonstrate comprehensive smoke suppression relative to the unmodified PA66/GF composite.

Overall, the above results indicate that increasing the ADP/MPP loading can continuously improve the LOI and effectively reduce the peak heat release rate, whereas the incorporation of DOPO–MA–oligomerPA6 can shorten the afterflame time, increase the LOI, and further reduce heat release compared with P1 at lower total additive loadings. Among the formulations, P3 exhibited the lowest pHRR, P8 achieved the highest LOI and the shortest afterflame time, and P7 exhibited the lowest THR. Considering that P7 required 30% less flame-retardant additive than P3 while maintaining a relatively high LOI and low cumulative heat release, the formulation containing 4 phr of the synergist achieved a favorable balance between additive loading and flame-retardant performance.

#### 3.2.4. Electrical Safety

Table 9 summarizes the glow-wire results. PA66, P0, and P1 failed at 700 °C. P2 passed at 700 °C, whereas P3 passed up to 750 °C, indicating an improvement with higher ADP/MPP loading. At a fixed ADP/MPP loading of 10 parts, the highest passing temperatures for P4, P5, P6, P7, and P8 were 725, 750, 750, 775, and 775 °C, respectively. P5 thus reached the same highest passing temperature as P3 at a total additive loading of 12 rather than 20 parts. P7 and P8 passed the 775 °C test at total loadings of 14 and 15 parts, respectively, corresponding to a reported GWIT of 800 °C for both formulations. The CTI was 500 V for PA66, P0, P1, and P2, but 600 V for P3. P4 and P5 also had CTI values of 500 V; at oligomer loadings of 3–5 parts, P6–P8 reached 600 V. P6 therefore achieved the same CTI as P3 at 13 rather than 20 parts total additives, a reduction of 35%. These results show that the oligomer-containing system meets both reported glow-wire and tracking-resistance targets at lower additive loadings.

#### 3.2.5. Overall Performance

A comprehensive analysis of structural characterization, morphological observations, and property measurements indicates that the overall performance of PA66/GF composites depends on an appropriate balance between the components and their loadings. GF significantly improved the mechanical properties and HDT of PA66, but the LOI of P0 decreased to 21.4%, and P0 did not achieve a UL-94 rating. Although increasing the ADP/MPP loading improved flame retardancy, it also reduced the tensile strength and HDT, indicating that simply increasing the loading of conventional flame retardants makes it difficult to balance flame retardancy and thermomechanical performance.

DOPO–MA–oligomerPA6 contains both phosphorus-containing structures and oligomeric polyamide segments, providing a structural basis for its involvement in flame retardancy and its interactions with the PA66 matrix. FT-IR results showed that the composites retained the characteristics amid absorption bands of PA66, while FESEM observations revealed no obvious large-scale agglomeration or phase separation. The synergistic formulations exhibited relatively good ductility and impact toughness, although some loss of tensile strength remained. Although the crystallinity of P4–P8 generally decreased, their HDT values were all higher than that of P1. This improvement may be related to the effects of the oligomeric polyamide segments on interfacial interactions and molecular chain mobility, but further verification is required.

As the synergist loading increased from 1 to 5 phr, the temperature corresponding to the maximum mass-loss rate increased from 447.0 °C to 467.8 °C, the residual mass at 700 °C generally increased, and the LOI increased from 28.1% to 39.2%. Compared with P1, P6–P8 exhibited lower pHRR and THR values, along with higher residual masses after combustion. These results suggest that synergists may improve flame retardancy by modifying thermal decomposition behavior and promoting the formation of solid residues. However, because thermogravimetric analysis and combustion testing involve different thermal environments, and the residues contain GF and inorganic components, an increase in residual mass alone is insufficient to demonstrate an enhanced barrier effect of the char layer.

The different formulations exhibited distinct advantages in terms of flame-retardant performance. P3 exhibited the lowest pHRR, P8 achieved the highest LOI and residual mass at 700 °C, whereas P7 exhibited the lowest THR and achieved a UL-94 V-0 rating, a GWIT of 800 °C, and a CTI of 600 V. Notably, improvements in tracking resistance may also involve changes in surface conductive pathways, and the underlying mechanisms require further investigation.

Overall, P7 exhibited a favorable balance of properties. Compared with P3, P7 achieved a 30% reduction in the total flame-retardant additive loading, a decrease in THR from 48 to 44 MJ·m^−2^, and an increase in HDT from 136.9 °C to 178.8 °C, while maintaining favorable mechanical properties. Further increasing the synergist loading improved the LOI but did not lead to additional improvements in THR or HDT. Therefore, optimizing the blending ratio of DOPO–MA–oligomerPA6 and ADP/MPP represents a feasible strategy for balancing flame retardancy, electrical safety, and thermomechanical performance in PA66/GF composites.

## 4. Conclusions

In this study, a phosphorus-containing polyamide oligomer, DOPO–MA–oligomerPA6, was designed and synthesized and subsequently combined with ADP and MPP to prepare flame-retardant PA66/GF composites. The successful synthesis of the target product was confirmed by ^1^H NMR, FT-IR, and GPC analyses, and its number-average molecular weight was determined to be 10,984 g·mol^−1^. The incorporation of DOPO–MA–oligomerPA6 effectively improved the self-extinguishing performance of the composites and reduced heat release at a lower total flame-retardant additive loading. When the loadings of ADP/MPP and the synergist were 10 and 4 phr, respectively, P7 achieved a UL-94 V-0 rating and an LOI of 36.1%, while its THR decreased to 44 MJ·m^−2^, the lowest value among all the tested formulations. Meanwhile, the GWIT reached 800 °C, and the CTI reached 600 V, indicating that this combined flame-retardant system could simultaneously improve flame retardancy and electrical safety.

Under these formulation conditions, the composite exhibited a HDT of 178.8 °C, a tensile strength of 95.0 MPa, a notched impact strength of 8.7 kJ·m^−2^, and a flexural modulus of 8984 MPa. Compared with the conventional formulation containing 20 phr of ADP/MPP, P7 achieved a 30% reduction in the total flame-retardant additive loading while exhibiting a lower THR, a higher HDT, and favorable mechanical properties, demonstrating a better balance of overall performance. When the synergist loading was further increased to 5 phr, the LOI of P8 increased to 39.2%, whereas its THR increased and its HDT decreased. These results indicate that synergist loading affects different properties to varying degrees and that an appropriate blending ratio is essential for balancing flame retardancy, electrical safety, and thermomechanical properties. This study provides experimental evidence to support the formulation design of halogen-free flame-retardant PA66/GF composites with low additive loadings and balanced overall performance.

Future research should combine pyrolysis gas analysis with the characterization of the morphology, composition, and structure of combustion residues to further elucidate the roles of DOPO–MA–oligomerPA6 and ADP/MPP in the gas and condensed phases and clarify their synergistic flame-retardant mechanisms. On this basis, the effects of synergist molecular weight, phosphorus content, and blending ratio on thermal decomposition behavior, interfacial interactions, and overall performance should be systematically investigated to establish relationships between molecular structure and material properties. Furthermore, the long-term performance stability of the composites under thermal aging, hygrothermal exposure, and prolonged electrical stress should be evaluated. The migration and exudation behavior of the flame-retardant components, as well as the processing adaptability of the composites, should also be investigated to provide further support for their potential applications in electrical and electronic insulating structural components.

## Figures and Tables

**Table 1:** Formulations of the flame-retardant PA66/GF composites.

Mass Fraction (100 phr of PA66/GF Matrix)				
Sample	PA66 (Mass Fraction)	GF (Mass Fraction)	Flame Retardant FR ADP:MPP = (8:2)/Mass Fraction	DOPO–MA–oligomerPA6 (Mass Fraction)
PA66	100	—	—	—
P0	75	25	—	—
P1	75	25	10	—
P2	75	25	15	—
P3	75	25	20	—
P4	75	25	10	1
P5	75	25	10	2
P6	75	25	10	3
P7	75	25	10	4
P8	75	25	10	5

Note: The PA66/GF base formulation was fixed at 100 parts by mass, with a PA66:GF mass ratio of 75:25. ADP/MPP and DOPO–MA–oligomerPA6 were added per 100 parts by mass of the PA66/GF base.

**Table 2:** FT-IR spectral information of the flame-retardant synergist DOPO–MA–oligomerPA6.

Wavenumber/cm^−1^	Functional Group Assignment
3302.1	Hydrogen-bonded amide N–H stretching vibration
2939.9, 2868.7	Asymmetric and symmetric stretching vibrations of aliphatic –CH_2_– groups
1715.7	Mainly attributed to the C=O stretching vibration of carboxyl groups
1633.6	Amide I band, primarily attributed to C=O stretching vibrations
1541.5	Amide II band (coupled N–H in-plane bending and C–N stretching vibrations), overlapping with aromatic C=C skeletal stretching vibrations of DOPO
1468.9	Primarily attributed to –CH_2_– scissoring vibrations, possibly overlapping with aromatic ring vibrations
1432.3	Possibly associated with DOPO aromatic ring skeletal vibrations and P–aryl-related vibrations
1366.4	CH_2_ deformation and coupled chain-segment vibrations
1196.4	P=O stretching vibrations or P–O–C/aromatic C–O stretching vibrations (possibly overlapping with absorption bands of the matrix)
919.2	Possibly associated with P–O–Ar-related vibrations in the DOPO structure

**Table 3:** Molecular-weight characteristics of DOPO–MA–oligomerPA6.

Sample	Mn (g mol^−1^)	Mw (g mol^−1^)	Mz (g mol^−1^)	Mw/Mn
DOPO–MA–oligomerPA6	10,984	28,223	48,063	2.569

**Table 4:** Mechanical properties of the flame-retardant PA66/GF composites.

Sample	Tensile Strength (MPa)	Elongation at Break (%)	Notched Charpy Impact Strength (kJ m^−2^)	Unnotched Charpy Impact Strength (kJ m^−2^)	Flexural Modulus (MPa)
PA66	77.1 ± 4.5	100.2 ± 2.5	3.7 ± 0.3	No break	2500 ± 21
P0	120.6 ± 5.1	5.6 ± 0.3	8.7 ± 0.3	52.2 ± 1.2	8903 ± 34
P1	110.3 ± 3.2	5.1 ± 0.4	8.4 ± 0.2	38.6 ± 2.2	9610 ± 26
P2	95.1 ± 1.3	5.2 ± 0.3	8.1 ± 0.1	36.2 ± 3.3	9058 ± 34
P3	90.2 ± 2.6	4.9 ± 0.4	7.9 ± 0.4	35.7 ± 4.1	8597 ± 13
P4	99.8 ± 4.7	5.3 ± 0.2	8.5 ± 0.4	38.5 ± 2.1	9321 ± 27
P5	96.2 ± 3.3	5.5 ± 0.3	8.5 ± 0.3	38.2 ± 3.1	9122 ± 35
P6	95.6 ± 4.1	5.6 ± 0.1	8.6 ± 0.2	38.0 ± 3.2	9026 ± 44
P7	95.0 ± 2.8	5.7 ± 0.2	8.7 ± 0.4	38.7 ± 2.4	8984 ± 35
P8	93.5 ± 3.1	6.0 ± 0.5	8.8 ± 0.5	38.8 ± 3.3	8800 ± 36

**Table 5:** Assignments and comparison of the main FT-IR absorption bands of different PA66/GF composites.

Wavenumber/cm^−1^	Functional Group Assignment	PA66/GF	PA66/GF (Traditional Flame Retardants)	PA66/GF (Traditional Flame Retardants and DOPO–MA–oligomerPA6)
3300	Amide N–H stretching vibration	Discernible	Weak	Discernible
2940, 2870	Aliphatic CH_2_ asymmetric and symmetric stretching vibrations	Discernible	Weak	Discernible
1633	Amide I band, primarily associated with C=O stretching vibrations	Distinct	Weak	Distinct
1541	Amide II band, primarily associated with coupled N–H in-plane bending and C–N stretching vibrations	Distinct	Weak	Distinct

Note: The descriptions of absorption intensity in the table refer only to the apparent absorption depth in the current figure. Since the spectra were vertically offset and neither consistent normalization nor uniform measurement conditions were provided, no quantitative comparison of peak intensities was performed.

**Table 6:** Thermal parameters and HDT (1.82 MPa) of PA66 and PA66/GF composites.

Sample	*T_c_* (°C)	Δ*H_c_* (J g^−1^)	*T_m_* (°C)	Δ*H_m_* (J g^−1^)	*X_C_* (%)	*T*_5%_ (°C)	*T_max_* (°C)	Residue at 700 °C (%)	HDT (°C)
PA66	208.2	54.7	260.2	43.8	23.3	430.2	472.6	2.0	70.3
P0	234.5	32.2	261.5	40.9	29.0	449.3	484.0	26.7	215.7
P1	229.8	31.2	258.8	39.5	30.8	385.0	424.4	24.8	142.3
P2	229.1	23.7	262.1	32.2	26.3	372.0	420.8	24.5	138.0
P3	226.8	17.4	260.8	22.7	19.3	379.3	426.3	28.5	136.9
P4	224.1	32.4	259.1	23.3	18.3	404.4	447.0	27.8	165.1
P5	222.4	30.8	261.4	22.0	17.5	402.3	459.1	33.0	177.2
P6	222.3	28.0	262.3	20.7	16.6	402.0	460.9	31.1	173.5
P7	223.6	25.8	259.6	16.8	13.6	401.4	461.8	33.1	178.8
P8	218.7	22.4	259.7	13.8	11.3	399.9	467.8	34.1	167.5

Note: *T_c_*, crystallization peak temperature; Δ*H_c_*, crystallization enthalpy; *T_m_*, melting peak temperature; Δ*H_m_*, melting enthalpy; *X_c_*, Crystallinity; *T*_5%_, temperature at 5% mass loss; *T_max_*, temperature at the maximum mass-loss rate; residue at 700 °C, residual mass percentage; HDT, heat deflection temperature.

**Table 7:** UL-94 vertical burning results and limiting oxygen index (LOI) of the PA66/GF composites.

Sample	t_1_ (s)	t_2_ (s)	t_1_ + t_2_ (s)	Molten Dripping	UL-94 Rating	LOI (%)
PA66	>60	—	—	Yes; cotton ignited	—	24.1
P0	>60	—	—	Yes; cotton ignited	—	21.4
P1	35	22	57	Yes; cotton ignited	V-2	28.5
P2	8	10	18	No; cotton not ignited	V-0	33.6
P3	5	4	9	No; cotton not ignited	V-0	37.2
P4	13	12	25	No; cotton not ignited	V-1	28.1
P5	6	5	11	No; cotton not ignited	V-0	31.4
P6	2	4	6	No; cotton not ignited	V-0	33.8
P7	1	2	3	No; cotton not ignited	V-0	36.1
P8	1	0	1	No; cotton not ignited	V-0	39.2

**Table 8:** Cone calorimetry results for PA66 and the PA66/GF composites.

Sample	TTI (s)	pHRR (kW m^−2^)	Time to pHRR (s)	THR (MJ m^−2^)	Residual Mass (%)
PA66	57	692	87	63	0.06
P0	43	732	95	61	22.69
P1	41	325	72	53	31.23
P3	50	165	121	48	38.46
P6	38	252	51	48	35.54
P7	40	270	53	44	39.06
P8	44	252	54	49	39.21

**Table 9:** Glow-wire test results and comparative tracking index (CTI) of PA66 and the PA66/GF composites.

Sample	700 °C	725 °C	750 °C	775 °C	800 °C	CTI (V)
PA66	×	×	×	×	×	500
P0	×	×	×	×	×	500
P1	×	×	×	×	×	500
P2	√	×	×	×	×	500
P3	√	√	√	×	×	600
P4	√	√	×	×	×	500
P5	√	√	√	×	×	500
P6	√	√	√	×	×	600
P7	√	√	√	√	×	600
P8	√	√	√	√	×	600

Note: √, passed at the indicated glow-wire test temperature; ×, failed. Specimen thickness: 1.5 mm. According to the GWIT definition in GB/T 5169.13-2024, the reported GWIT is generally 25 °C above the highest test temperature meeting the specified non-ignition criterion (or 30 °C in the applicable higher-temperature range). P7 and P8 passed at 775 °C; their corresponding GWIT is therefore reported as 800 °C. The value 775 °C denotes the highest passing test temperature, not the GWIT.

## Data Availability

The original contributions presented in this study are included in the article. Further inquiries can be directed to the corresponding authors.

## Abbreviations

The following abbreviations are used in this manuscript:

PA66	Polyamide 66
GF	Glass fiber
PA66/GF	Glass-fiber-reinforced polyamide 66
GWIT	Glow-wire ignition temperature
CTI	Comparative tracking index
DOPO	9,10-Dihydro-9-oxa-10-phosphaphenanthrene-10-oxide
DOPO–MA–oligomerPA6	Phosphorus-containing polyamide oligomer synthesized in this work
DOPO–MAH	DOPO–maleic anhydride adduct (intermediate)
MAH	Maleic anhydride
ADP	Aluminum diethylphosphinate
MPP	Melamine polyphosphate
PA6	Polyamide 6
PDI	Polydispersity index
UL-94	UL-94 vertical burning test
LOI	Limiting oxygen index
HDT	Heat deflection temperature
P(DH-co-MA)	Poly(DOPO-hydroxyethyl acrylate-co-maleic anhydride)
MCA	Melamine cyanurate
AR	Analytical reagent grade
ε-CPL	ε-Caprolactam
FR	Flame retardant
^1^H NMR	Proton nuclear magnetic resonance spectroscopy
FT-IR	Fourier-transform infrared spectroscopy
GPC	Gel permeation chromatography
HFIP	Hexafluoroisopropanol
PMMA	Poly(methyl methacrylate)
FESEM	Scanning electron microscopy
TGA	Thermogravimetric analysis
DTG	Derivative thermogravimetry
DSC	Differential scanning calorimetry
TTI	Time to ignition
PHRR	Peak heat release rate
tPHRR	Time to peak heat release rate
THR	Total heat release
HRR	Heat release rate
SPR	Smoke production rate
pSPR	Peak smoke production rate
TSR	Total smoke release
